# Sensing prior constraints in deep neural networks for solving exploration geophysical problems

**DOI:** 10.1073/pnas.2219573120

**Published:** 2023-06-01

**Authors:** Xinming Wu, Jianwei Ma, Xu Si, Zhengfa Bi, Jiarun Yang, Hui Gao, Dongzi Xie, Zhixiang Guo, Jie Zhang

**Affiliations:** ^a^School of Earth and Space Sciences, University of Science and Technology of China, Hefei, 230026 China; ^b^Mengcheng National Geophysical Observatory, University of Science and Technology of China, Hefei 230026, China; ^c^School of Earth and Space Sciences, Institute of Artificial Intelligence, Peking University, Beijing 100871, China

**Keywords:** geophysical problems, deep neural networks, prior constraints, deep learning

## Abstract

One of the key objectives in geophysics is to characterize the subsurface through the process of analyzing and interpreting geophysical field data that are typically acquired at the surface. Data-driven deep learning methods have enormous potential for accelerating and simplifying the process but also face many challenges, including poor generalizability, weak interpretability, and physical inconsistency. We present three strategies for imposing domain knowledge constraints on deep neural networks (DNNs) to help address these challenges. The first strategy is to integrate constraints into data by generating synthetic training datasets through geological and geophysical forward modeling and properly encoding prior knowledge as part of the input fed into the DNNs. The second strategy is to design nontrainable custom layers of physical operators and preconditioners in the DNN architecture to modify or shape feature maps calculated within the network to make them consistent with the prior knowledge. The final strategy is to implement prior geological information and geophysical laws as regularization terms in loss functions for training the DNNs. We discuss the implementation of these strategies in detail and demonstrate their effectiveness by applying them to geophysical data processing, imaging, interpretation, and subsurface model building.

## Significance

We present three possible strategies to effectively incorporate geological and/or geophysical constraints into deep neural networks (DNNs). They help address the main challenges of poor generalizability, weak interpretability, and physical inconsistency that are commonly faced when applying DNNs to solve geophysical problems. In discussing each of the three strategies, we provide examples of applications to demonstrate their effectiveness.

Geophysical problems typically involve dealing with large amounts of spatial and temporal data that generally are obtained from various sources and show high heterogeneity. Deep neural networks (DNNs) can play an important role in this because of their versatility in extracting information, merging multisource information, universal approximation, and high expressivity. During the past decade, DNNs have been widely used in various geophysical research areas (1–5), including seismology (6–14), atmospheric science (15–17), and planetary and space science (18–20). DNNs have been particularly intensively studied in exploration of geophysics to accelerate and advance the entire workflow of data processing (21–24), tomography (25–29), forward modeling (30–35), migration (36–40), velocity model building (41–47), and interpretation (48–53).

However, there still remain many challenges in employing DNNs to solve geophysical problems. One challenge is the lack of sufficient training datasets in the geophysics field, wherein most existing data are unlabeled. Moreover, the limited available labels of field geophysical data are often highly subjective or biased (1) because the ground truth of geophysical research objects is typically unknown or unreliable. The insufficient and inaccurate labels of training datasets limit the performance of DNNs, especially of those based on supervised learning. Another challenge is the variety of field geophysical data that the trained DNNs are applied to. In practice, the geophysical data fed into a trained DNN model can significantly differ from the training data because of the variation in data acquisition (e.g., spatial and temporal resolution, frequencies, and survey geometries), noise, and geophysical processing workflows. Together, these challenges lead to a poor generalizability of trained DNN models when applied to process or interpret geophysical datasets. Some other challenges, including low interpretability and poor physical consistency (2), which arise from the common theoretical weakness of DNNs, may be amplified in geophysical applications owing to the high complexity and uncertainty of geophysical data.

One potential approach to improve model generalizability, interpretability, and physical consistency is to impose domain knowledge constraints (prior geological information and/or geophysical laws) on the DNNs. Reichstein et al. (2) suggested to integrate contextual cues in deep learning to further improve its predictive ability by developing hybrid model- and data-driven networks coupling domain knowledge and data statistics learning. Some authors suggested designing specialized network architectures to incorporate prior constraints for seismic waveform inversion (54) and geomechanical log prediction (55). Di et al. (53) and Kong et al. (56) imposed constraints on DNNs by inputting manually interpreted results and physics-based features into the networks, respectively. Other authors (57–60) integrated physical constraints in DNNs through loss functions for training. The significance of imposing prior constraints on DNNs is highly recognized, and this topic is recommended as the next research focus in DNNs for geosciences (2, 5). However, the strategies to implement such constraints are incompletely discussed, and some are unexplored. It is worthwhile to provide a general recipe to follow for imposing geological and/or geophysical constraints on DNNs.

**Fig. 1.**
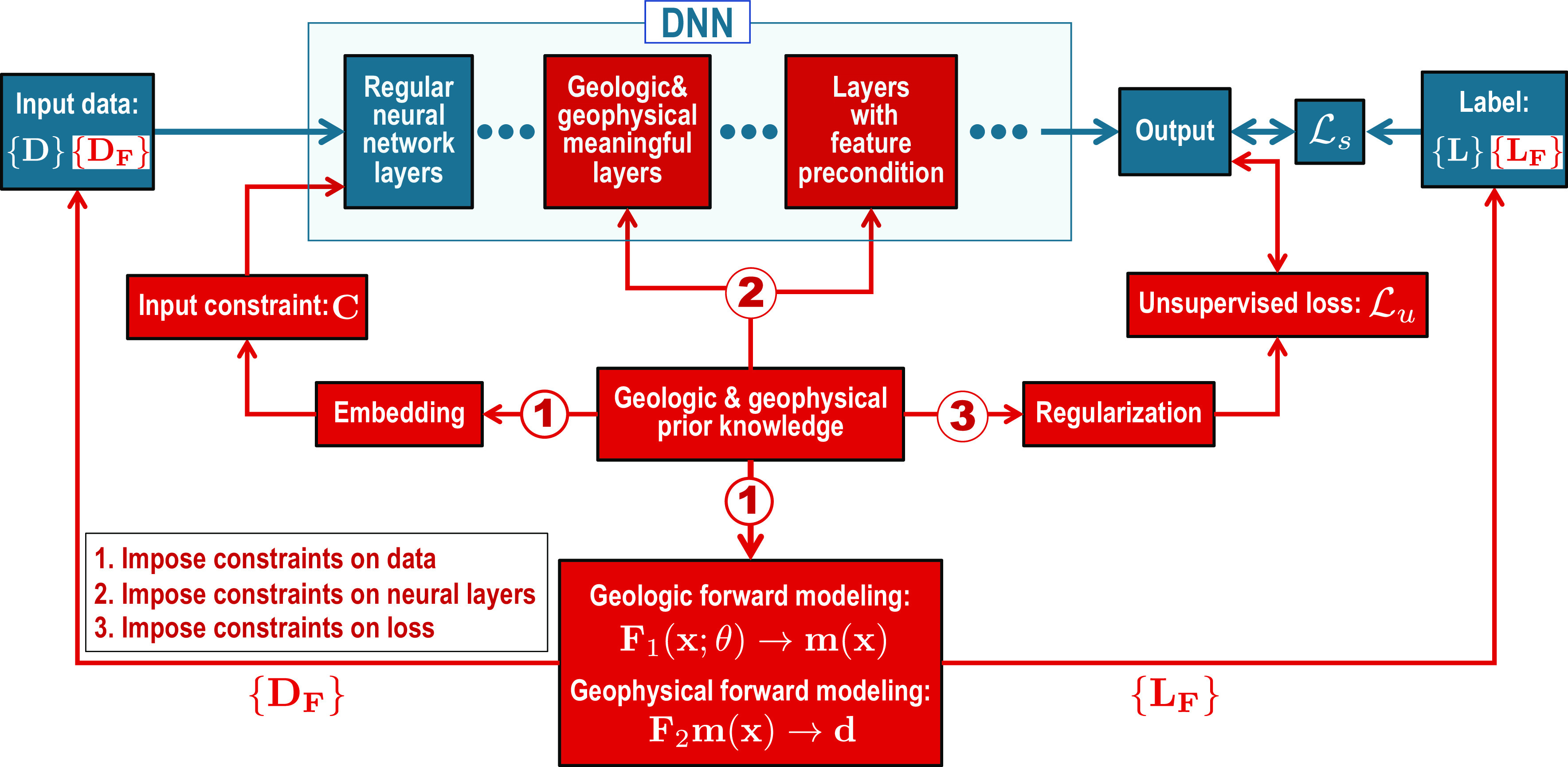
Three strategies for integrating prior geological and geophysical constraints (in red) into a regular DNN framework (in blue). The first strategy is to integrate prior knowledge into data including input data with embedded knowledge and synthetic training data simulated by geology- and geophysics-informed forward modeling. The second strategy is to impose constraints directly on the network including defining geologically and geophysically meaningful layers and preconditioning the feature maps in the network. The third strategy is to define a knowledge-based loss function for training the network.

We present a comprehensive discussion of three general strategies (Fig. 1) to incorporate geological and geophysical constraints into deep learning methods to improve their accuracy and reliability in solving geophysical problems. We first show that integrating prior knowledge into training data and input data of neural networks is an effective method to impose constraints on data-driven deep learning. We then present an even more effective approach to impose constraints by defining custom layers with prior knowledge in a neural network and preconditioning feature maps calculated in the network. The prior constraints imposed in this manner are considered hard constraints because they are satisfied not only in the training process but also in the inference step. Finally, we discuss the most straightforward method to impose constraints on DNNs by defining loss functions with prior knowledge. In explaining each of the three strategies, we provide examples of their applications in geophysical data processing, imaging, interpretation, and subsurface model building. However, we believe that these strategies can not only be applied to these specific topics but also to more general geophysical problems.

## Imposing Constraints on Data

Deep learning is a data-driven method, and it is generally agreed that data and their characteristics determine its upper limit (61). In this section, we demonstrate that the performance of a DNN is significantly affected by the features included in the datasets to train the network and the data fed into the network. This indicates that we can effectively impose prior constraints on a DNN by embedding prior knowledge or expected features into the training or input data.

### Embedding Prior Knowledge in Training Data.

The lack of labeled training data remains a big challenge in applying DNNs in the geoscience field because the subsurface ground truth is typically unknown and manual labeling is highly subjective and labor-intensive. One method to address this challenge is to generate synthetic training datasets with labels by using various numerically geological and geophysical forward modeling methods (6, 43, 52, 62–67).

In this approach, the ground truth labels are automatically generated, eliminating the need for human labeling. In addition, this method is flexible in that modeling parameters are randomly chosen to generate numerous training datasets with diverse features. Here, we consider forward modeling as an approach to embed prior knowledge into training datasets, which are then used to train a DNN to extract the embedded knowledge from the datasets. Such a trained DNN is expected to extract the similar knowledge from field data that resemble the synthetic data.

#### General workflow.

**Fig. 2.**
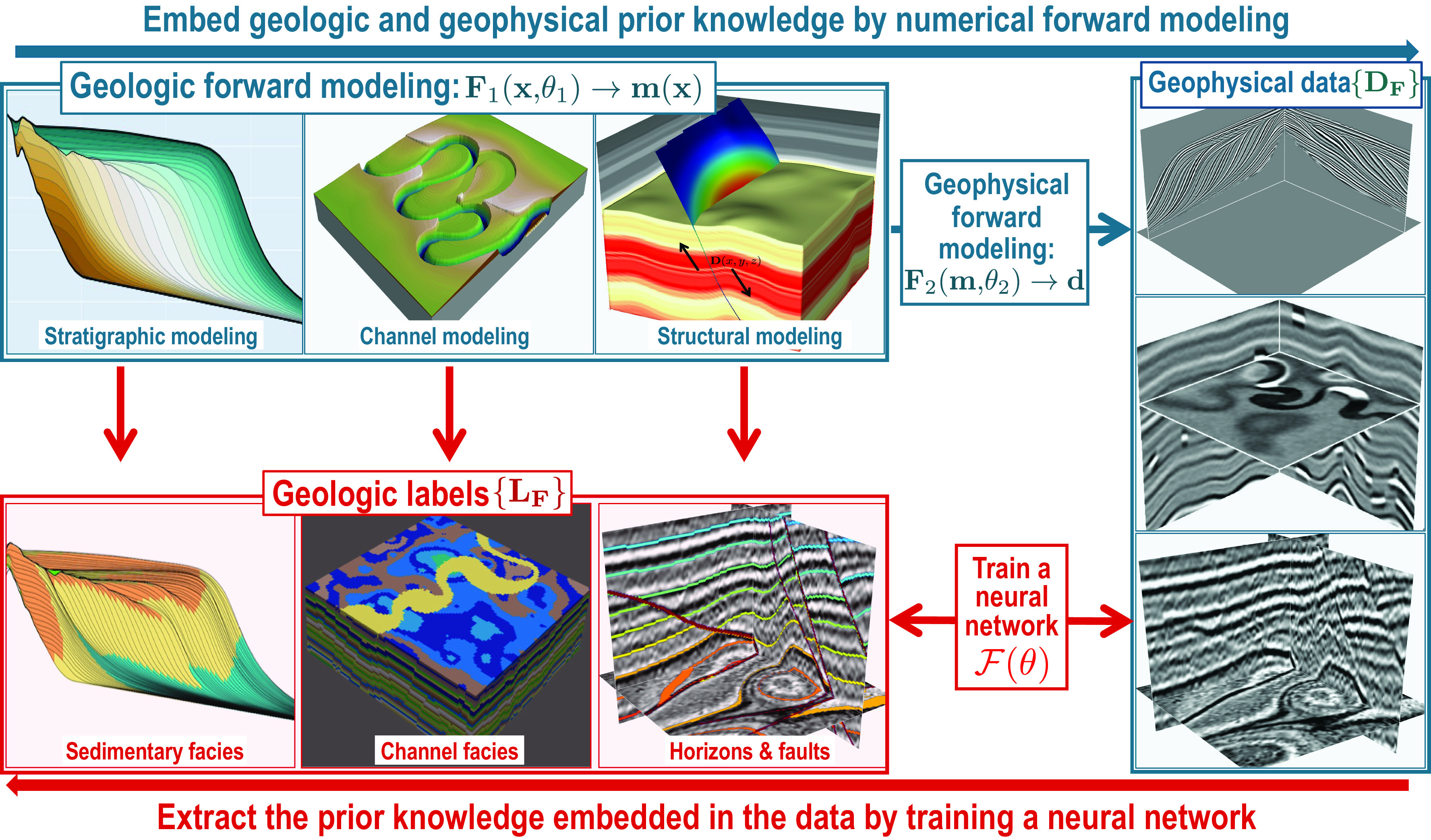
A strategy to impose constraints on a DNN by creating synthetic training datasets based on prior knowledge. In this strategy, we first automatically generate numerous synthetic training datasets by numerical forward modeling which embeds prior geological and geophysical knowledge in the data. Using the generated training data, a neural network is then trained or constrained to extract the prior knowledge embedded in the data.

Most geophysical problems involve extracting geological features or knowledge from geophysical data. Therefore, preparing training datasets for geophysical problems typically includes generating geophysical datasets and corresponding geological labels. Fig. 2 shows a general workflow to generate geophysical training datasets and corresponding labels. First, numerical geological forward modeling is performed to obtain digital geology models **m(x)**, which can be expressed as follows:

[1] $$\begin{array}{c} m\left(x\right)=F_{1}\left(x,\theta_{1}\right), \end{array}$$

where **x** denotes the model space and **F**_1_ represents geological forward processes, such as stratigraphic (68, 69), channel (70, 71), and structural (67) forward modeling, as shown in the *Upper Left* panels in Fig. 2. The forward simulation processes **F**_1_ are typically operators or equations that are carefully formulated with prior knowledge of the geology. The ***θ***_1_ represents a set of modeling parameters that are randomly selected from all possible options to generate numerous and diverse models. Through forward modeling, diverse geological properties or features are embedded in the models. The labels of the geological features or information of interest, such as sedimentary facies, channel facies, horizons, and faults, in the model space can be automatically defined, as shown in the lower left panels in Fig. 2. These geological labels {**L**_**F**_} are used to supervise the training of DNNs.

The simulated geological models can be converted to models of geophysical properties, such as densities, velocities, and impedances, by following prior rules. Therefore, using the simulated models **m**, we can perform geophysical forward modeling to simulate geophysical data **d** as follows:

[2] $$\begin{array}{c} d=F_{2}\left(m,\theta_{2}\right), \end{array}$$

where **F**_2_ represents geophysical forward modeling processes or operators, such as wave propagation and convolution, and ***θ***_2_ denotes a set of modeling parameters randomly selected from all possible options to generate numerous and diverse geophysical data {**D**_**F**_}. The simulated geophysical data shown in the right panel in Fig. 2 are 3D seismic images that are computed by convolving reflectivity models with Ricker wavelets. These three images, from top to bottom, contain geologically meaningful information on stratigraphic geometries, channel features, and folding and faulting structures, respectively. These image features correspond to the geological knowledge (like the geological labels in the lower left panels in Fig. 2) embedded in the geological models that are used to simulate the images. The goal is to use the pair of training datasets {**D**_**F**_} and {**L**_**F**_} to train a DNN that can automatically and accurately extract the geological knowledge embedded in the geophysical data.

This workflow can be summarized as a two-step process of first performing forward simulation with well-defined equations or prior knowledge to generate data and then training DNNs to achieve an inverse mapping ℱ(***θ***) of knowledge from the generated data:

[3] $$\begin{array}{r} F_{1}(x,\theta_{1})\to m(x)\to F_{2}(m(x),\theta_{2})\to d.\\ \tilde{m}(x)\leftarrow F(\theta )\leftarrow d \end{array}$$

Inverse mapping of geological knowledge from geophysical data is typically more challenging than the forward process and is sometimes hardly defined by equations. DNNs are powerful in statistically approximating such complex mappings by learning from a large number of training datasets. Note that the forward modeling of the training data and the training process of a DNN can be executed simultaneously so that the data generation can be adaptively stopped when the training converges.

Sometimes we may want to use a DNN to approximate geophysical forward modeling to accelerate the data simulation. In this case, we do not need to explicitly generate data to train the DNN. Instead, we can include physical forward modeling as part of the DNN architecture or training loop (3) where physical equations or operators are implemented in the loss function to serve as a physical supervision for training the DNN. This is actually a classic type of unsupervised learning or physics-informed neural networks, which we discuss in more detail in the section on imposing constraints on loss functions.

**Fig. 3.**
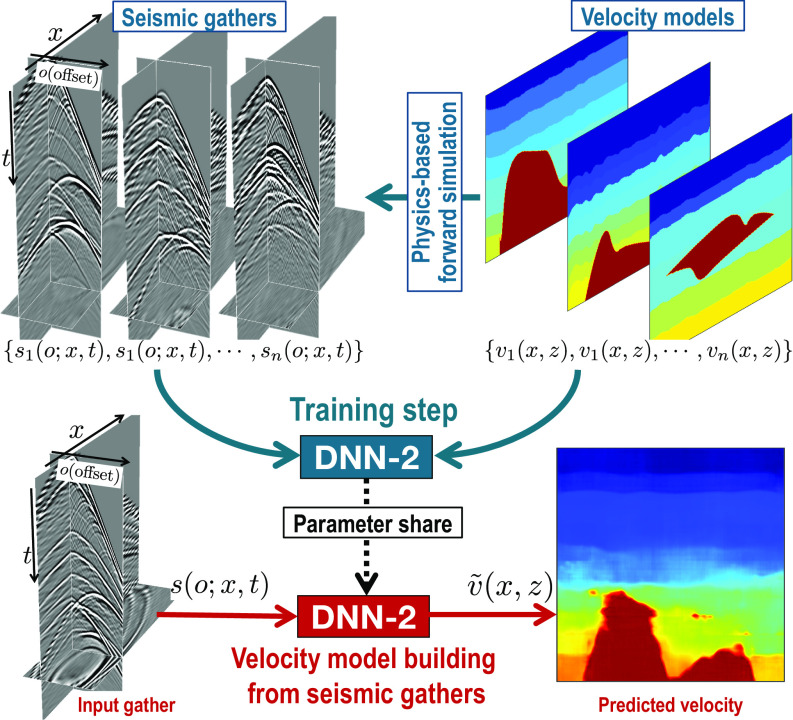
An example of integrating prior knowledge into training datasets. Based on the physical relationships between velocity models and seismic gathers, we can perform physics-based forward simulation to create numerous synthetic training datasets from which DNNs learn direct velocity model building from recorded raw seismic gathers e.g., refs. 42, 43, 46, and 45.

Various DNN models, trained by synthetic datasets, have been successfully applied to solve multiple geophysical problems including seismic denoising (7, 22, 72, 73), seismic migration (36, 40), velocity model building (25, 29, 42, 43, 45, 74, 75), impedance inversion (62, 76), and seismic interpretation (52, 71, 77, 78). As shown in Fig. 3, synthetic training datasets, generated by geophysical forward simulation, have been used to train DNNs (42, 43, 45, 46) to direct build velocity models $\tilde{v}\left(x,z\right)$ from recorded raw seismic gathers *s*(*o*; *x*, *t*) in the domain of space (*x*), time (*t*), and offset (*o*). In these processes, prior domain knowledge (i.e., the physical relationship between the velocity models and simulated data) is embedded in the synthetic training datasets by physics-based forward modeling. DNNs are constructed to learn from the training datasets to infer the direct mapping from data (semblance cubes or seismic gathers) to velocity models. These methods provide a potentially ideal means to directly image the subsurface from recorded geophysical data without the need to solve computationally expensive and ill-posed inversion problems as in conventional geophysical imaging workflows. However, they have been rarely applied to complex field examples with desirable results. This is probably because the size and richness of the synthetic training datasets are insufficient and the field examples can strongly differ from the training datasets in terms of geological background, data acquisition (including spatial and temporal resolution, frequencies, and survey geometries), noise, and processing errors. Improving the diversity of synthetic training datasets is essential, and some authors (42) suggested to use generative adversarial networks (79) for this purpose.

#### Fault interpretation with synthetic training datasets.

To better explain how the diversity and authenticity of synthetic training datasets affect the performance of a DNN model, we take convolutional neural network (CNN)-based seismic fault detection as an example. We consider fault detection as a binary image segmentation problem and use the CNN model proposed in ref. 52 for segmentation. The model was trained using the synthetic datasets (*SI Appendix*, Fig. S1*A*) published along with (52), and a balanced cross-entropy loss function was used to optimize the network parameters. This model has been successfully applied to multiple field examples (52) but failed to compute a clean or continuous fault detection in our example (*SI Appendix*, Fig. S1*D*). Moreover, this model completely missed the detection of the large fault highlighted by the red arrows in *SI Appendix*, Fig. S1*C*.

To improve the performance of the CNN model, we increased the diversity of the synthetic training datasets in three aspects. First, in the reported synthetic seismic images (52), all faults were mainly featured as reflection discontinuities caused by fault displacement. The actual fault highlighted by the red arrows in *SI Appendix*, Fig. S1*C*, however, appears as consistent and strong reflection features, which significantly differ from the synthetic fault features. Therefore, we specifically built fault surfaces whose hanging-wall and foot-wall block impedances appeared high contrasts to generate continuous and strong reflection features at the faults highlighted by the red arrows in *SI Appendix*, Fig. S1*B*. Second, seismic fault detection is typically sensitive to discontinuity features such as noise and data processing artifacts or errors. However, the reported synthetic seismic images (52) contained only simple random noise, which is insufficient to approximate the discontinuity and noisy features in field seismic images. Therefore, we extracted real noise and artifacts from multiple field seismic images using a structure-oriented smoothing filter and added the extracted features to the clean synthetic seismic images to make them more realistic (*SI Appendix*, Fig. S1*B*). Third, we further increased the diversity of fault patterns in the synthetic training datasets. In particular, we included more low-dip angle faults and corresponding dragging features. We retrained the CNN model with the updated training datasets and applied the retrained model to the field seismic image (*SI Appendix*, Fig. S1*C*). We thus obtained an updated fault detection result (*SI Appendix*, Fig. S1*E*) in which the fault features are substantially cleaner and more continuous than those in *SI Appendix*, Fig. S1*D*. Moreover, the large fault (highlighted by red arrows in *SI Appendix*, Fig. S1*C*), misdetected in *SI Appendix*, Fig. S1*D*, was consistently detected in the updated result in *SI Appendix*, Fig. S1*E*.

Training dataset diversity is important for training a well-generalized DNN model. However, in practice, it is difficult to prepare a completely diverse training dataset, especially in the geophysics field. The above example demonstrates that when the trained model does not work well for specific test data, we may first check whether the training dataset is sufficiently diverse to include the features or patterns appearing in the test data or not. If not, we may consider updating and enriching the training dataset based on prior knowledge or information about the test data, including visual features, sampling rates, and frequency components. Embedding prior knowledge or information in the training datasets is an effective means to guide a DNN model to reasonably perform predictions according to expectancy. However, this is an indirect manner to impose prior knowledge, as desired features need to be first simulated in the synthetic data and the DNN model needs to be retrained using these data to learn the features. In addition, some actions in simulating realistic features (e.g., adding noise and artifacts) in synthetic data are useful but may not be physically or geologically meaningful.

### Embedding Prior Constraints in Input Data.

Another method to impose constraints on data is to properly embed or encode prior knowledge or information as tensors that can be directly fed into the DNN model. The advantage of this approach is that we can implicitly impose constraints on the DNN model in the inference step so as to effectively introduce human interactions when applying the model to the test data. Some authors have shown that inputting prior information, such as subsurface structural features (80), low-frequency data features (81), and initial velocity models (29), into DNNs is helpful to improve their robustness for seismic full waveform inversion. Below, we present two examples to illustrate this in detail.

#### Relative geologic time (RGT) estimation with interpreted horizons.

Estimating an RGT volume from a 3D migrated seismic image is a volumetric method for interpreting a full volume of seismic horizons and building stratigraphic models (82). However, estimating an accurate or geologically reasonable RGT volume remains challenging in cases where the input seismic image is complicated by growing faults, unconformities, heavy noise, or poor imaging quality. Moreover, owing to the limited seismic resolution or imaging errors, a seismic reflection does not necessarily follow a chronostratigraphic horizon or surface with constant geologic time. In such cases, none of the data-driven methods can obtain a geologically reasonable RGT result or horizon by exactly fitting or following the seismic reflections. Therefore, some horizons, manually interpreted based on prior geological knowledge, are typically required to be used as constraints or guidance to obtain reasonable RGT estimations in the above-mentioned complicated cases.

**Fig. 4.**
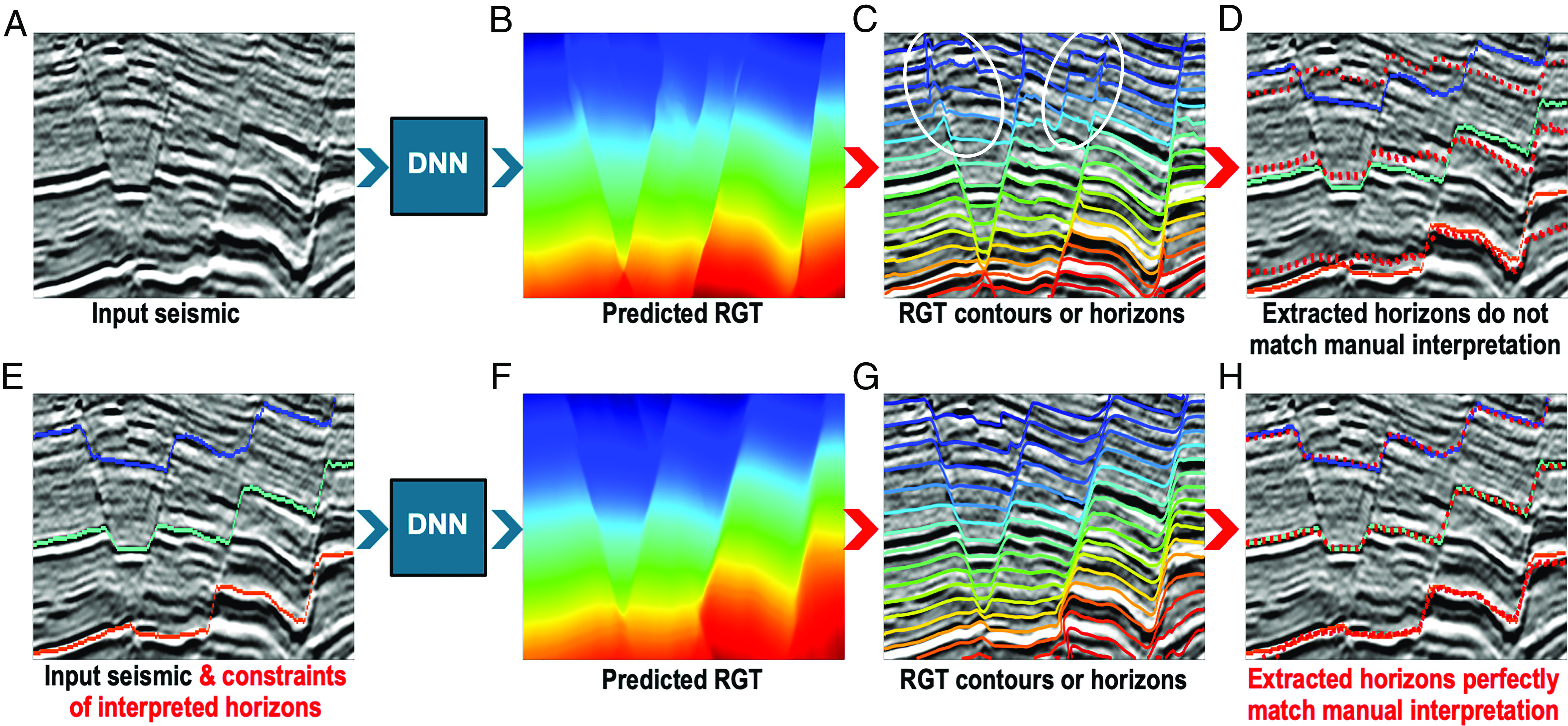
DNN-based RGT estimation and horizon extraction without (*Upper* panels) and with (*Lower* panels) inputting constraints of manually interpreted horizons. Without the input of horizon constraints, the contours (horizons) extracted from the estimated RGT result, did not accurately follow seismic reflections, especially in the areas highlighted by the white ellipses in (*C*).

The *Upper* panels in Fig. 4 show an example of deep-learning-based RGT estimation (Fig. 4*B*) from an input seismic image (Fig. 4*A*) complicated by growing faults. This DNN was designed using a vision transformer (83) and trained with synthetic datasets generated by structural and geophysical modeling (67). A hybrid loss function of mean square error and multiscale structural similarity was used to optimize the network parameters. The trained DNN model worked well in multiple field examples and produced a visually reasonable RGT result with sharp fault features in the example shown in Fig. 4*B*. However, the horizons, extracted as contours of the estimated RGT result, did not accurately follow seismic reflections, especially in the areas crossing the faults, which are highlighted by the white ellipses in Fig. 4*C*. The estimated horizons (red dashed curves in Fig. 4*D*), especially the *Uppermost* one, did not follow the corresponding manually interpreted horizons (blue, cyan, and orange curves).

To improve the performance of the DNN model, we retrained it using the same synthetic datasets but with a two-channel input of a seismic image and a constraint image of interpreted horizons. The constraint image was of the same size as the seismic image and was defined as a mask with zeros everywhere, except at the positions near the interpreted horizons. The values near an interpreted horizon were defined as the average of the heights (vertical coordinates) of the horizon. By embedding the interpreted horizons into the input in this manner, we were able to effectively impose the constraints of the horizons on the DNN and obtain a substantially more reasonable result, as shown in the *Lower* panels in Fig. 4.

#### Implicit structural modeling with faults.

**Fig. 5.**
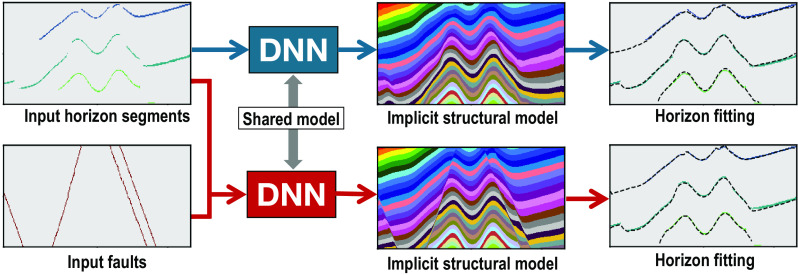
DNN-based implicit structural modeling without (an empty fault mask, *Upper* panels) and with (*Lower* panels) inputting constraints of a fault mask (*Lower* *Left* image). Both models fit the shared input horizons, but the lower one with the prior constraints of input faults generates sharp fault structures.

Building a structure model typically requires or involves frequent and intensive human interactions to update the model. When structural modeling is implemented through DNNs (84–86), the most convenient means to incorporate human interactions is to embed them into the inputs of the DNNs. Fig. 5 shows an example of CNN-based implicit structural modeling (86) with a two-channel input horizon and fault segments. As shown in the *Upper* panels in Fig. 5, with the input of horizon segments and an empty fault mask, the DNN predicted a reasonable implicit structural model (upper middle panel in Fig. 5), with the folding structures of the layers fitting the input horizons. With the knowledge of the subsurface faults, we input the known faults as a binary mask combined with the horizons to the same DNN and obtained an updated structural model (*Lower Middle* panel in Fig. 5) that contained sharp fault structures corresponding to the input faults but still fitted the folding structures of the input horizons. As shown in the *Right* panels in Fig. 5, the horizons (black dashed curves) extracted from the two predicted structural models both matched with the input horizon segments (colored curves) well. This indicates that a ground truth of the subsurface is generally missing and a solution based on limited data or prior information (e.g., the horizons only) of the subsurface is typically nonunique.

In most geoscience problems, the solution must be continuously updated by integrating gradually updated data and prior knowledge. Deep learning provides a convenient means to merge all types of input data and prior information (that may be from various sources and modalities) to make a comprehensive prediction. Embedding prior information into the input of DNNs is a convenient means to impose constraints on or implement human interactions in the trained DNNs in the inference step to improve their generalizability and obtain geologically or geophysically reasonable results. Moreover, the inference step of a trained DNN model is highly efficient; we can quickly or even immediately obtain an updated result by modifying the inputs of the model.

## Integrating Constraints into Network

We discussed that embedding prior knowledge into the training or input data is an effective method to impose constraints on DNNs. However, constraints imposed in this manner are not guaranteed to be satisfied by the output of a DNN in the inference step. We present two methods to impose hard constraints on a DNN by defining custom layers and implementing feature preconditioning with prior knowledge in the DNN architecture.

### Defining Custom Layers.

**Fig. 6.**
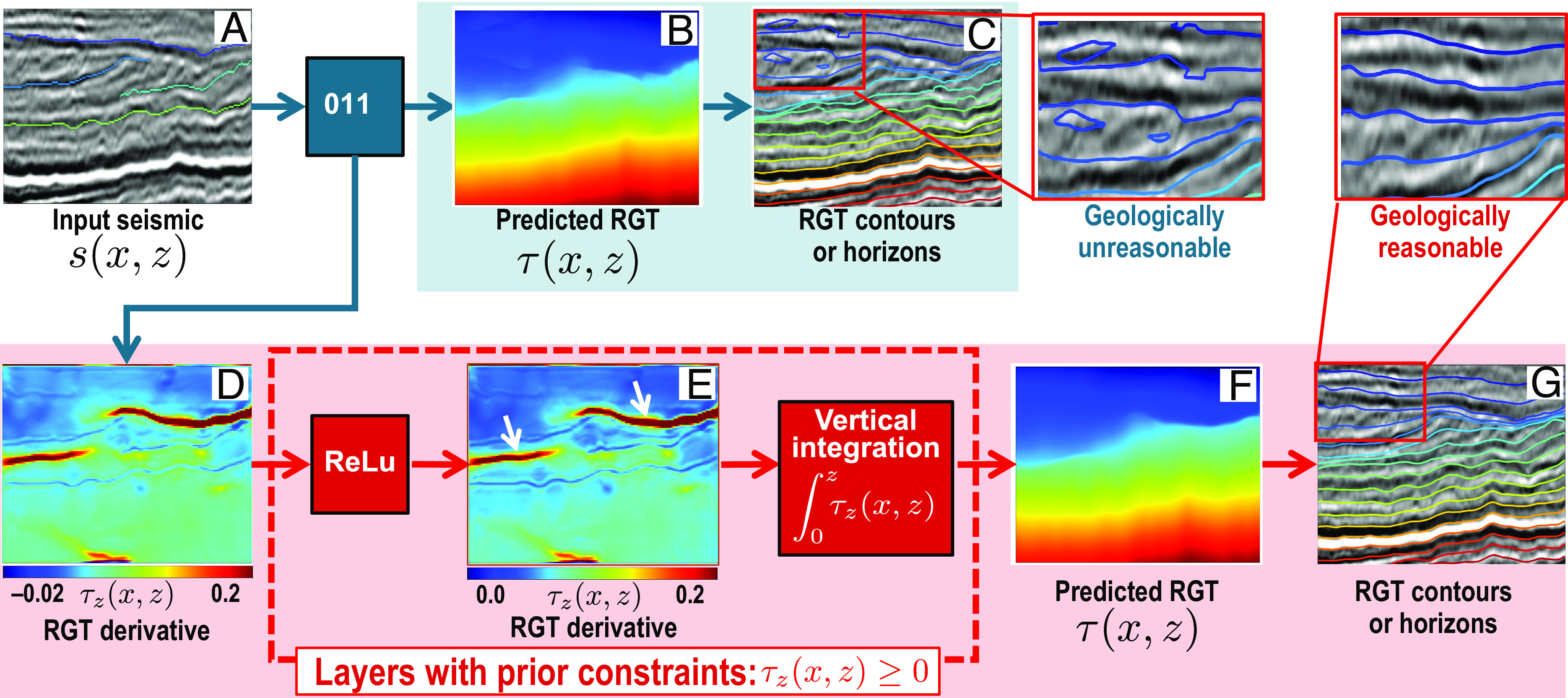
DNN-based RGT estimation and horizon extraction without (*Upper* panels) and with (*Lower* panels) custom neural layers that are implemented with prior constraints. The custom neural layers not only ensure a final reasonable RGT estimation but also yield an intermediate result of RGT derivatives that could be used as a byproduct to highlight unconformities as denoted by the white arrows in (*E*).

**Fig. 7.**
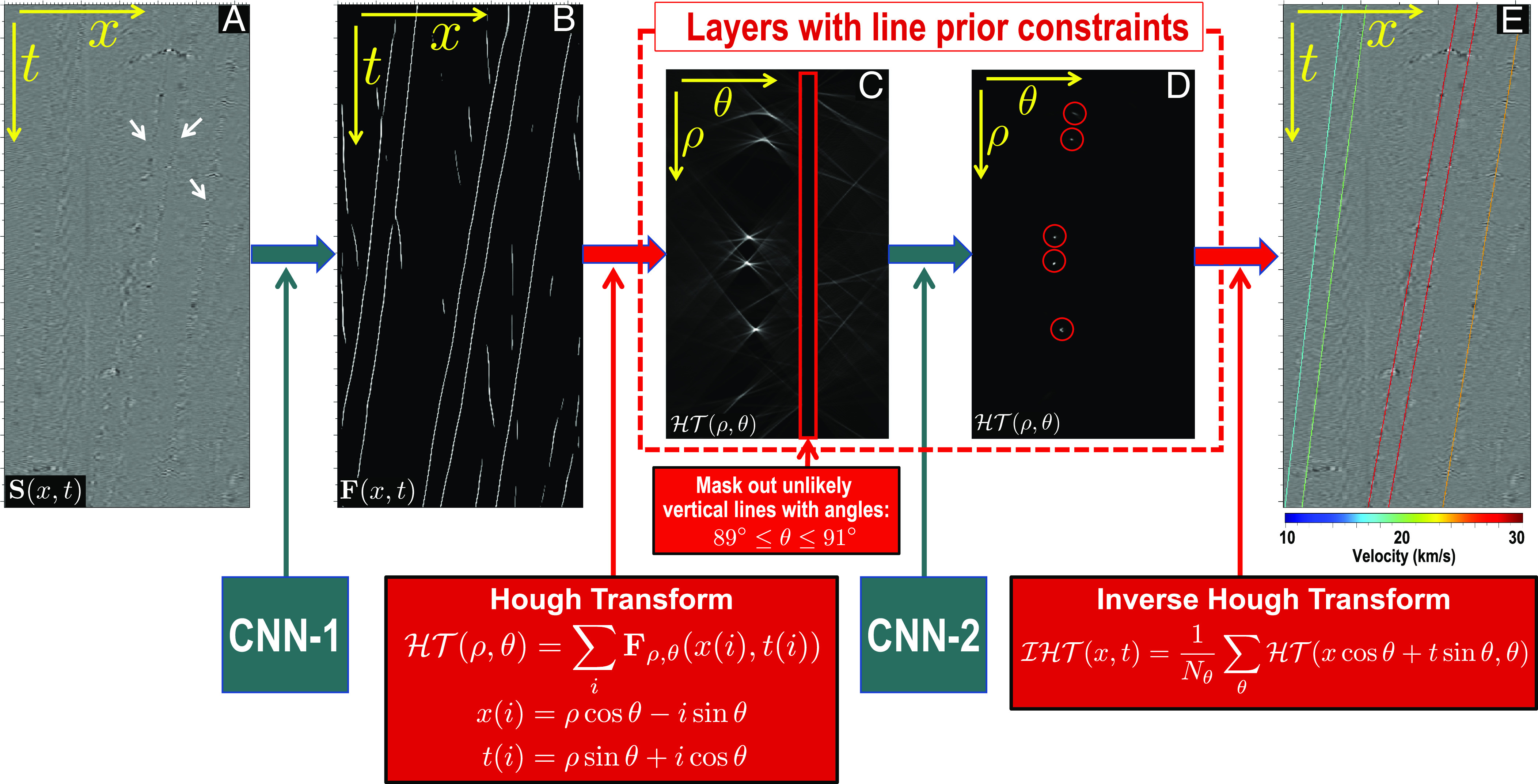
Custom neural layers of Hough transform (HT) are implemented in the DNN for automatic vehicle tracking and speed estimation from distributed acoustic sensing data. With these custom layers, the prior constraint that vehicle traces are locally linear is effectively imposed on the DNN to ensure complete line detections as shown in (*E*). In addition, it is straightforward to estimate the vehicle speed (color of the lines in (*E*) in the HT domain (*ρ*, *θ*) where the located *θ* can be directly converted to the speed. The white arrows in (*A*) denote linear vehicle traces recorded in the raw DAS data while the red circles in (*D*) highlight focused dots that correspond to the detected linear traces in the HT domain.

To explain the idea of designing custom neural network layers with prior constraints, we use two examples of RGT estimation (Fig. 6) and vehicle trace extraction from distributed acoustic sensing (DAS) data (Fig. 7).

#### RGT estimation with physical layers.

The upper panels in Fig. 6 show an example of RGT (*τ*(*x*, *z*)) estimation from the input of a seismic image (*s*(*x*, *z*)) and some manually interpreted horizons by using the same DNN model as in Fig. 4. From the estimated RGT map, we extracted contours to obtain the horizons in Fig. 6*C*, most of which accurately followed seismic reflections. However, we also observed circular horizons (in the red boxed area in Fig. 6*B*), which are geologically unreasonable as circular geologic layers are rarely observed in the subsurface. In most cases (without structural inversion), the geologic time of layers increases vertically. This prior knowledge can be implemented as a hard constraint to eliminate the unreasonably circular contours (horizons) in the RGT estimation.

To implement the constraint that ensures a vertically increasing RGT result, we assumed that the DNN predicts an intermediate result of the vertical RGT derivative (*τ*_*z*_(*x*, *z*)) as shown in Fig. 6*D*. We applied an activation function, rectified linear unit (ReLu), to the RGT derivative

[4] $$\begin{array}{c} f\left(\tau_{z}\right)=\max\left(0,\tau_{z}\right)=\tau_{z}^{+}, \end{array}$$

which yielded a nonnegative map of derivatives $\tau_{z}^{+}\left(x,z\right)$ (Fig. 6*E*). We finally computed a final RGT map *τ*(*x*, *z*) by integrating the derivative in the vertical direction, as follows:

[5] $$\begin{array}{c} \tau \left(x,z\right)=\int_{0}^{z}\tau_{z}^{+}\left(x,z\right)dz. \end{array}$$

Such an integration can be simply implemented with the cumsum function in Python. This block (red dashed box in Fig. 6) of ReLu and integration layers implements physical operators with prior constraints and contains no training parameters. However, this block is also part of the overall DNN architecture and is executed in both the training and inference steps to ensure a final RGT result (Fig. 6*F*) with vertically increasing values. The contours (horizons) extracted from this RGT map are no longer circular, and all accurately follow seismic reflections (Fig. 6*G*). Note that for the intermediate RGT derivative computation in the constrained DNN, we did not explicitly supervise it with a derivative map during the training. Instead, we trained both DNNs with or without the block of constraint layers using the same RGT labels. After training, the constrained DNN automatically computes the intermediate RGT derivative map so that an integration from which can yield the final RGT map. Moreover, this derivative map could be used as a byproduct to highlight unconformities and faults with relative high values as denoted by the white arrows in Fig. 6*E*.

#### Vehicle trace analysis in DAS data.

Another example is vehicle trace analysis in 2D DAS data (Fig. 7*A*). The data **S**(*x*, *t*) were acquired by a highly sensitive DAS array near an urban road where vibrations due to vehicles and noise sources were recorded. The lateral axis *x* of the data represents the DAS array direction, and the vertical axis *t* denotes the time at which the vibrations were received. The linear features (denoted by white arrows in Fig. 7*A*) represent vehicle motions recorded by the DAS array. The dips of the linear features indicate the speeds of the vehicles when passing along the DAS array. The tasks of extracting the linear traces and estimating their dips are complicated by heavy noise, which was also recorded by the sensitive DAS array.

We present a deep-learning–based method with prior constraints implemented in its DNN architecture to automatically analyze vehicle traces in noisy DAS data. The entire DNN architecture (Fig. 7) contains a CNN block (CNN-1), followed by a block of layers with prior constraints (included in the red dashed box). The first CNN block (CNN-1) is a regular Unet (87) trained to compute a line detection map **F**(*x*, *t*) (Fig. 7*B*) from the input DAS data **S**(*x*, *t*) (Fig. 7*A*) in the space–time domain. The CNN-1 block is able to detect the vehicle traces (the long lines in Fig. 7*B*) within the input data but also yields some noisy linear features (short segments). In addition, obtaining the individual line instances (corresponding to different vehicles) and estimating their dips (corresponding to vehicle speeds) from such a line detection map remains challenging.

We propose the addition of another block of constrained layers, following the line detection, to solve the above problems. This block consists of a Hough transform, another CNN block (CNN-2), and an inverse Hough transform. The Hough transform maps the line detection **F**(*x*, *t*) from the space–time domain into the Hough domain ℋ𝒯(*ρ*, *θ*), where the vertical axis represents the radius *ρ* and the lateral axis denotes the angle *θ*. In this domain, we can easily filter out the unlikely vertical lines with angles 89° ≤*θ* ≤ 91° (corresponding to slowly moving or static objects) by simply masking out the area denoted by the red box in Fig. 7*C*. Perfect lines in the space–time domain will be points in the Hough domain. However, the image features in the original domain (Fig. 7*B*) are not perfectly linear, resulting unfocused radiant patterns of features in Fig. 7*C*. Therefore, another simplified Unet (CNN-2) is used to compute better focused point features (Fig. 7*D*) in the Hough domain, where we can easily locate the positions of five points (*ρ*_*i*_, *θ*_*i*_),*i* = 1, 2, ⋯, 5. These points are transformed back to the original space–time domain to obtain five perfect lines in Fig. 7*E*. The colors of the lines represent the vehicle speeds that are converted from the angles *θ*_*i*_ located in the Hough domain. Three regression loss functions were constructed with the outputs of the CNN-1 (Fig. 7*B*), CNN-2 (Fig. 7*D*), and the final result (Fig. 7*E*), respectively, to jointly train the entire network.

The Hough transform and its inverse transform are inner components of the entire neural network and are implemented as matrix–vector operations to enable gradient back propagation during the training of the network. The matrices of Hough transform and its inverse transform are predefined and contain no training parameters. By implementing the transforms as components of the network, the prior knowledge of “linear vehicle traces” is successfully imposed as a hard constraint on the network. Similar to the previous example of RGT estimation, this constraint is imposed in both the training and inference steps and the output is ensured to satisfy the prior constraint.

### Feature Map Preconditioning.

A more straightforward method to integrate constraints into a network is to apply prior knowledge-based preconditioning to the feature maps that are calculated in the neural network layers, especially the decoder layers near the output. Such preconditioning can be performed using smoothing filters or any other operators to modify the values in the feature maps and make them consistent with the prior knowledge.

**Fig. 8.**
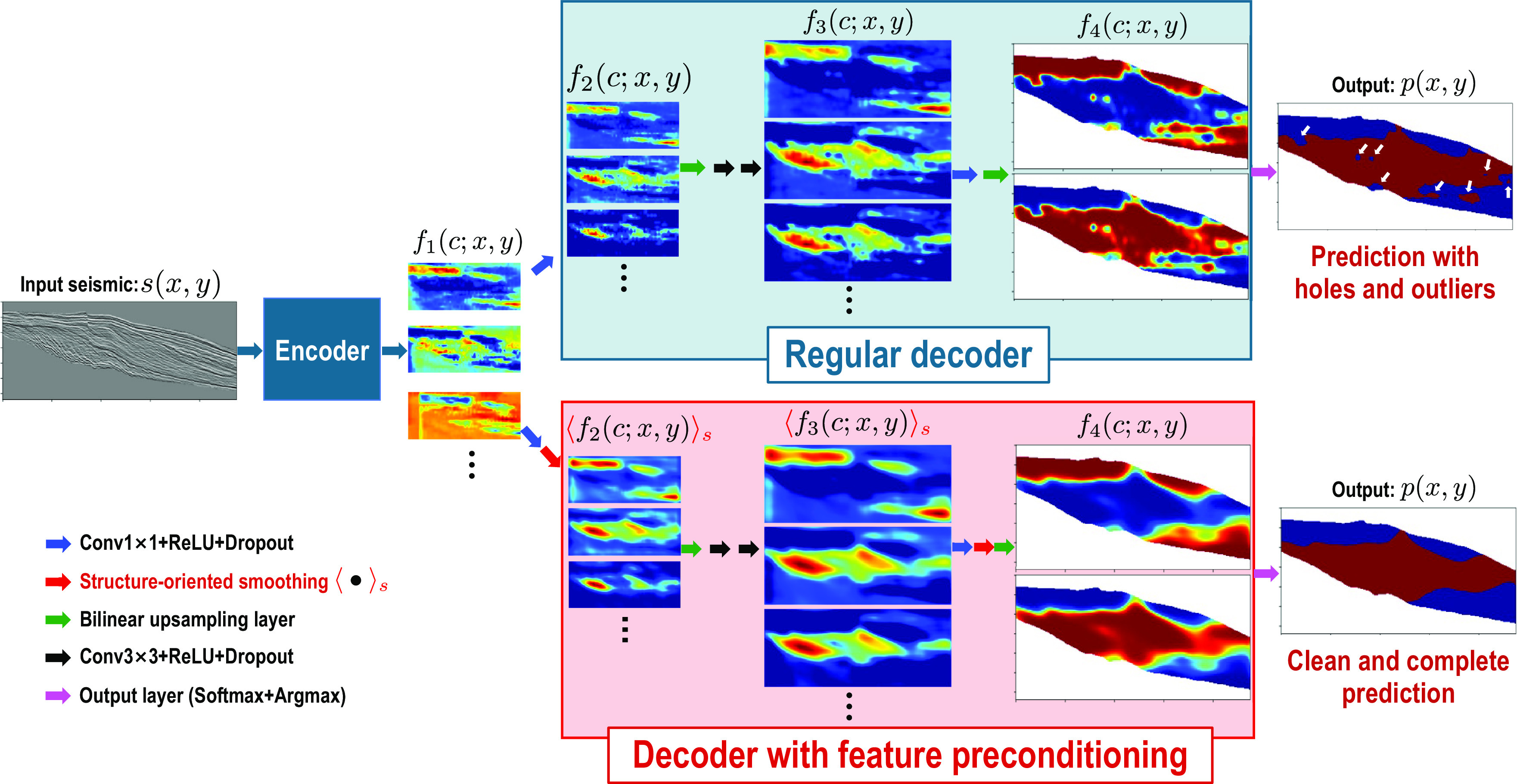
Example of imposing constraints on a DNN by applying preconditioning (*Lower-Right* branch) to feature maps calculated in the neural network layers, especially in the decoder layers near the output. The preconditioning here is applying structure-oriented smoothing to the feature maps, which is helpful to fill holes and eliminate outliers that are apparent in the seismic clinoform segmentation (*Upper-Right* image) without feature preconditioning.

Fig. 8 shows an example of seismic clinoform segmentation by using an encoder–decoder CNN architecture. The upper panels in Fig. 8 show some feature maps calculated in the *i*−th layer of a regular decoder:

[6] $$\begin{array}{c} f_{i}(c;x,y),\;i=1,2,3,4, \end{array}$$

where *c* represents the number of feature maps calculated at the *i*−th layer. From these sequentially computed feature maps, a final clinoform segmentation result *p*(*x*, *y*) is obtained, as shown in the upper right image in Fig. 8. The segmented clinoform area (denoted in red) is mostly accurate but contains some unreasonable holes and outliers, which are denoted by the white arrows.

One important observation is that the feature maps of the decoder layers strongly resemble the final output of clinoform segmentation. Some noisy features corresponding to the output holes and outliers can also be observed in the feature maps *f*_*i*_(*c*; *x*, *y*), especially when *i* = 2, 3. Based on these observations, we can apply structure-oriented smoothing ⟨ ⋅ ⟩_*s*_ kernels to reshape the feature maps, as follows:

[7] $${\langle f_{i}\left(c;x,y\right)\rangle }_{s},\;\;\;i=2,3.$$

This results in more continuous map features and the suppression of noise, as shown in *Lower* panels in Fig. 8. Note that we applied the smoothing to relatively small-scale feature maps to lower the computational cost. The idea to apply such smoothing was based on the prior knowledge that the spatial extension of the clinoform should be continuously aligned with seismic reflections. Therefore, we could use seismic structure information to design structure-oriented and spatially varying convolutional kernels to enhance the feature maps to yield a more reasonable clinoform segmentation result without holes or outliers (*Lower Right* image in Fig. 8).

We have provided some simple examples to illustrate the strategy of integrating prior constraints into networks by designing custom layers and applying feature preconditioning, which typically does not include any training parameters. This is the most effective strategy to impose prior constraints that are guaranteed to be satisfied during the inference step. However, implementing prior constraints (e.g., complex physical processes or operators) in the network is not straightforward, and related work in geophysics has been rarely published. More research on this type of strategy is encouraged and required in the geophysics field.

## Integrating Constraints into Loss Functions

Imposing prior constraints through loss functions is similar to implementing regularization terms in the objective functions for geophysical inversion problems. It has been widely studied, especially in semisupervised and unsupervised learning, and physics-informed neural networks (PINNs).

### Semisupervised and Unsupervised Learning.

The lack of labeled training datasets is a common challenge for applying deep learning to solve most geoscience problems. Semisupervised or unsupervised learning is a potential way to address this challenge by employing unsupervised loss functions to enable the use of unlabeled datasets and prior constraints for training a DNN.

*SI Appendix*, Fig. S2 shows a simple framework of semisupervised learning with supervised (ℒ_*s*_) and unsupervised (ℒ_*u*_) loss functions that are jointly used to optimize the network parameters. The supervised loss is typically built on only a small set of labeled data. The unsupervised loss ℒ_*u*_ integrates the large amount of unlabeled data into the training process based on prior knowledge or a consistency constraint of the predictions, which is essential for training a better generalized network model. When no labels are available for supervision, the supervised loss (*SI Appendix*, Fig. S2) is missing, which leads to an unsupervised learning fashion. Various semisupervised and unsupervised learning strategies (88, 89) have been proposed, which typically differ in the choice of the unsupervised loss functions. A detailed discussion of semisupervised/unsupervised learning and how to implement unsupervised loss is provided in *SI Appendix*.

Semisupervised learning has been widely used to solve geophysical problems (49, 58, 90–94). Based on a similar idea, physics-guided and data-driven hybrid training schemes (95–97) have been proposed to improve the robustness and generalizability of deep learning methods for geophysical inversion. In these schemes, physical guidance is introduced into the training step by defining the unsupervised loss ℒ_*u*_ based on geophysical laws. However, they differ from the PINNS discussed below in that they do not utilize automatic differentiation (98) to calculate the derivatives involved in the loss. Unsupervised learning can discover hidden patterns in unlabeled data and has also been used for various tasks including seismic facies classification (50, 99, 100), seismic signal or waveform classification (11, 101–103), lithology classification (104–106), seismic migration (37, 39), and inversion (107, 108).

### PINNS.

Another representative approach to impose prior information or constraints on a neural network through loss functions is to build PINNs and train them with physics-informed loss functions that are constructed based on governing physical laws (e.g., partial differential equations (PDE)s). By minimizing physics-informed loss functions, PINNs can be trained to estimate results satisfying the governing physical laws. Although the trained networks are typically ignorant of the underlying physics, they can infer the solution space of complex governing physical equations owing to their capability of universal approximation and high expressivity (109, 110). PINNs can solve both forward and inverse problems involving PDEs and, therefore, have emerged as a hot topic in machine learning for scientific computing (111, 112). PINNs can be constructed with various network architectures (112), such as fully connected (109–111, 113), recurrent (114–117), and convolutional (118–121) neural networks.

**Fig. 9.**
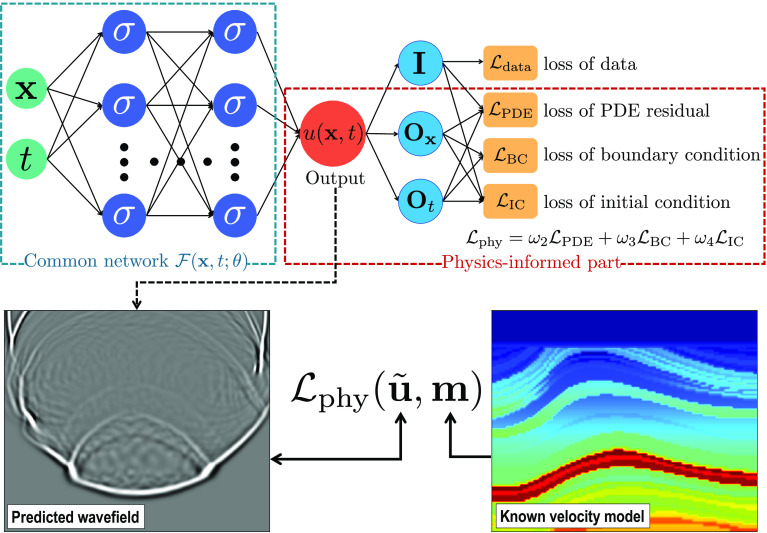
A simple framework of physics-informed neural network (PINN) (*Upper* panels) consists of a trainable common neural network ℱ(**x**, *t*; *θ*) and a physics-informed part without training parameters. Such a PINN can be employed for geophysical forward modeling problems (e.g., seismic wavefield simulation in *Lower* panels).

The upper panels of Fig. 9 show a simple PINN that is implemented with the architecture of fully connected neural network. This PINN framework consists of a first part of a common neural network and a second physics-informed part. Given the time *t* and space coordinates **x**, the network ℱ(*θ*) is trained to predict *u*(**x**, *t*)=ℱ(**x**, *t*; *θ*) that satisfies both measured data and some physical equations by minimizing a hybrid loss function as follows:

[8] $$\begin{array}{c} \arg\underset{\theta }{\min}\omega_{1}L_{\text{data}}(x,t;\theta )+\omega_{2}L_{\text{PDE}}(x,t;\theta ) \end{array}$$

[9] $$\;\;\;\;\;\;\;\;\;\;\;\;\;\;\;\;\;\;\;\;+\omega_{3}L_{\text{BC}}(x,t;\theta )+\omega_{4}L_{\text{IC}}(x,t;\theta ).$$

In the physics-informed part (within the dashed red box in Fig. 9), **O**_**x**_ and **O**_*t*_ represent physical operators applied to the prediction *u*(**x**, *t*) regarding the spatial coordinates **x** and time *t*, respectively. In PDEs, such operators are spatial and temporal derivatives scaled by predefined prior physical parameters (not trained). In PINNs, the derivatives are computed by automatic differentiation (98) which provides an accurate way to compute derivatives and avoids truncation or round-off errors appearing in numerical differentiation. **I** represents an identity operator, and the data loss ℒ_data_ is defined based on the similarity or difference between the prediction *u*(**x**, *t*) and the data measured at some specific times and spatial locations. The loss ℒ_PDE_ is the residual of the governing PDE that is defined by a combination of operations (**O**_**x**_ and **O**_*t*_) on the prediction *u*(**x**, *t*). The loss functions ℒ_BC_ and ℒ_IC_ enforce the prior boundary and initial conditions of the prediction, respectively.

The three physics-informed loss functions ℒ_PDE_, ℒ_BC_, and ℒ_IC_ incorporate the constraints of prior governing physical equations, boundary conditions, and initial conditions into the training process and make sure the prediction or solution satisfies the prior constraints. We can train the PINN by solely using the physics-informed loss functions without any labeled data, which can be actually considered as an unsupervised training strategy. If some measured data or carefully simulated data are available, we can use them to supervise the training process by through the data loss ℒ_data_ as well as the physics-informed losses, which can then be considered as a semisupervised training strategy. Such labeled data, although are typically limited, are helpful to speed up the convergence of the training process.

PINNs have been recently explored for solving geophysical forward modeling (31, 32, 34, 59, 122–125) and inversion (28, 47, 126–128), which involve intensive work on PDEs. Taking seismic modeling and inversion as an example, we can construct a basic framework for PINN-based geophysical forward modeling (*Lower* panels in Fig. 9) and inversion. In the forward modeling, a neural network (mostly constructed as a fully connected neural network) is trained to approximate a function ℱ(**x**, *t*, ***θ***) that can directly and continuously predict the wavefield $\tilde{u}\left(x,t\right)$ at an arbitrary time *t* and spatial position **x**. Based on the seismic wave equation, a known velocity model **m(x)** is used together with the predicted wavefield $\tilde{u}\left(x,t\right)$ to construct a physics-based loss function $L_{\;}\text{phy}\left(\tilde{u},m\right)$ for training the network until the wave equation is fitted or the loss minimization is converged. In the PINN-based inversion, another neural network ℱ(**x**, ***θ***) can be trained to directly infer a velocity model $\tilde{m}\left(x\right)$ that minimizes a loss function $L_{\;}\text{phy}\left(u,\tilde{m}\right)$ defined under the governing wave equation. In this inversion process, a forward modeling process is typically required to simulate the wave propagation in the subsurface to formulate the loss function, similar to what is done in traditional inversion schemes. Therefore, a pretrained forward modeling PINN is typically employed to simulate the wavefield data **u**, which is in turn used to construct the loss $L_{\;}\text{phy}\left(u,\tilde{m}\right)$ for training the PINN of inversion, as discussed in ref. 47 and 128. Considering that the forward modeling process is always coupled with the inversion, some authors (27, 35) suggested to jointly train the two PINNs for both forward modeling and inversion by linking the two networks via a common loss function. In this case, gradients are simultaneously back-propagated to both PINNs for updating their parameters during the training process.

In summary, PINNs provide another reasonable method to impose prior constraints on neural networks by constructing physics-informed loss functions based on physical equations to train the networks. It has been demonstrated in many applications that PINNs can effectively learn the solutions of the governing equations in a physics-informed manner with limited or even no labeled data. PINNs show some significant advantages over traditional methods for solving PDE problems. First, a PINN is a mesh-free algorithm that is able to avoid truncation and round-off errors owing to the grid-based discretization and is flexible to solve problems in arbitrary complex-geometry domains. Second, PINNs are able to directly solve nonlinear and complex problems without the need of committing to any assumptions, linearization, simplification, or local time-stepping (109), which are typically required by traditional solvers. Finally, the same PINN framework can be used to jointly solve both forward and inverse problems (112). However, there remain limitations in PINN-based solutions to geophysical forward modeling and inversion. First, training a PINN is quite tricky and computationally costly (it can be more expensive than traditional solvers), especially for predicting complex geophysical fields and earth models. Second, a PINN, pretrained for a certain PDE, can be seldom adapted or generalized to the variation of parameter settings (e.g., physical variables of the equation, initial and boundary conditions) for the same PDE. Third, forward modeling and inverse mapping functions, approximated by neural networks in PINNs, tend to make smooth predictions and therefore often seldom recover details or high-frequency features in the modeling and reversion results.

## Conclusions

Most types of DNNs have been intensively employed, especially in the last 5 y, for dealing with various types of geophysics tasks that involve extracting and picking key features, clustering, and classification, making predictions, forward simulation, and inversion for subsurface properties. DNNs have shown promising performance in several geophysical applications with high efficiency and accuracy but still face problems of weak interpretability, physical inconsistency, and poor generalizability in field applications. These problems of DNNs may be more obvious in geophysics than in many other areas because the lack of labeled training datasets and diverse inference datasets are substantially more serious in geophysics. In addition to continuing leveraging the latest deep learning techniques and properly reformulating geophysical problems in better deep learning fashions, future research on DNNs for geophysical problems should focus on integrating domain knowledge into DNNs to address the above problems and obtain better constrained DNN models.

We presented and demonstrated three general strategies to impose prior constraints on DNNs. The first one pertains to the training datasets and input data fed into the DNNs. Through geological and geophysical forward simulation followed by data augmentation, one can generate numerous and diverse synthetic training datasets, which directly solves the problem of missing labeled datasets and implicitly embeds domain knowledge into DNNs by using the simulated data to train them. By properly encoding prior knowledge as input channels (vectors or tensors), one can directly integrate constraints and, more importantly, introduce real-time human interaction or control into DNNs in the reference step. The second strategy is to construct specialized DNN architectures with custom layers that are designed based on physical operators without trainable parameters. As inner components of the DNN, these custom layers manipulate feature maps of the network (in both the training and inferring steps) to ensure that the output conforms to prior constraints. The third strategy is to integrate prior constraints into loss functions for training the DNNs, which is more flexible and straightforward to implement than the other two strategies. The second strategy imposes hard constraints on DNNs while the other two impose soft constraints, and all three are helpful to improve the generalizability, interpretability, and physical consistence of DNN models.

We believe that the successful application of DNNs to geophysical problems will require substantially more research on these three strategies. Research on the second strategy may be more challenging but is desirable. In addition, we expect research efforts to be made on topics including, but not limited to, building large-scale and diverse benchmark datasets (such as the SEG Advanced Modeling AI project), federated learning and active learning to fully use datasets onsite, dealing with the limitation of memory in field examples with high dimensions and large sizes, training a general large DNN model that can be transferred to various specific tasks in geophysics, and properly and completely utilizing or merging data and knowledge from multiple sources and modalities.

## Supplementary Material

Appendix 01 (PDF)

## Data Availability

Anonymized binary files for original data and their processing results included in the manuscript data have been deposited in Zenodo (https://doi.org/10.5281/zenodo.7326606).
