# Supplementary material for: Sensing prior constraints in deep neural networks for solving exploration geophysical problems
Source: Proc Natl Acad Sci U S A. 2023 Jun 1;120(23):e2219573120. doi: 10.1073/pnas.2219573120 (PMC10265955; doi:10.1073/pnas.2219573120)
Supplement: Supplementary file 1 — Appendix 01 (PDF) [file pnas.2219573120.sapp.pdf]

Supplementary Information for

Sensing prior constraints in DNNs for solving exploration geophysical problems

Xinming Wu, Jianwei Ma, Xu Si, Zhengfa Bi, Jiarun Yang, Hui Gao, Dongzi Xie, Zhixiang Guo, Jie Zhang

Jie Zhang.
E-mail: jzhang25@ustc.edu.cn;
Jianwei Ma.
E-mail: jwm@pku.edu.cn

This PDF file includes:

Supplementary text
Figs. S1 to S2

Supporting Information Text

Framework of semi-supervised learning

Fig. S2 shows a simple framework of semi-supervised learning with supervised ($\mathcal{L}_s$) and unsupervised ($\mathcal{L}_u$) loss functions that are jointly used to optimize the network parameters θ:

$$\min_{\theta} \sum_{x \in X_L, y \in Y_L} \mathcal{L}_s(x, y; \theta) + \sum_{x \in X_U} \mathcal{L}_u(x, \tilde{x}; \theta). \quad [1]$$

In this equation, X_L represents a small subset of data with labels Y_L, while X_U denotes a much larger subset of data without labelling. Using only the small set of labelled data to train a deep neural network (θ) by minimizing $\mathcal{L}_s$ may easily lead to over-fitting. The unsupervised loss $\mathcal{L}_u$ brings the large amount of unlabelled data X_U into the training process based on some prior knowledge or consistency constraint of the predictions, which would be essential for training a better generalized network model. Fig. S2 shows one typical type of consistency constraint where the predictions y_p and $\tilde{y}_p$, calculated by the same network from x and its perturbed or augmented version $\tilde{x}$, respectively, should be consistent. In this case, $\mathcal{L}_u$ is a measurement of the difference between the predictions y_p and $\tilde{y}_p$. The augmentation applied to x could be adding noise (**? ?**), rotation (**?**), flipping, resizing, calculating attributes (**?**) and so on. For the rotated, flipped, or resized $\tilde{x}$, we need to undo the corresponding augmentation of the prediction $\tilde{y}_p$ before comparing it to y_p.

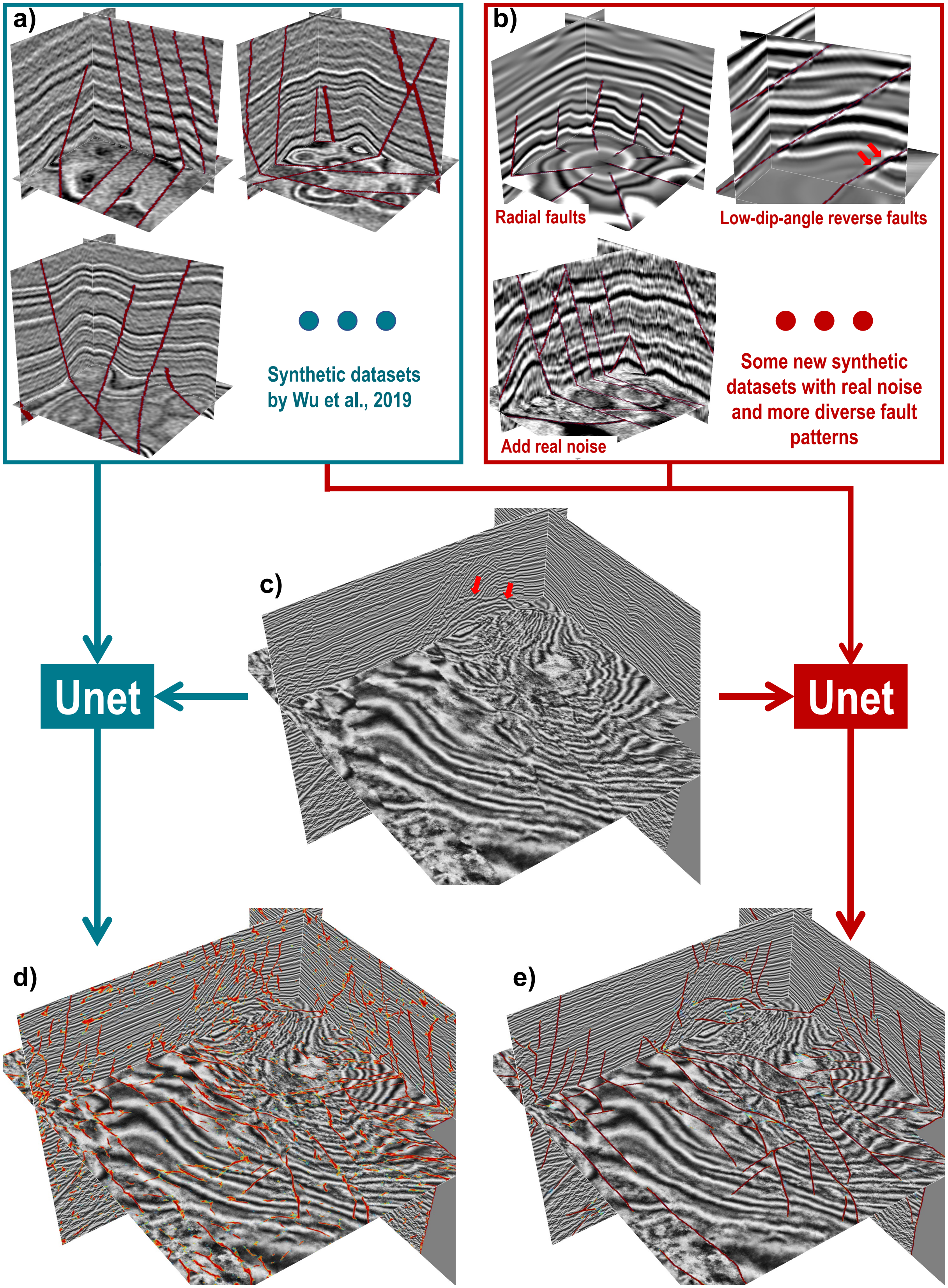

Fig. S1. Train a better generalized DNN model for seismic fault detection with more diverse and realistic training datasets that are numerically simulated based on geological and geophysical knowledge.

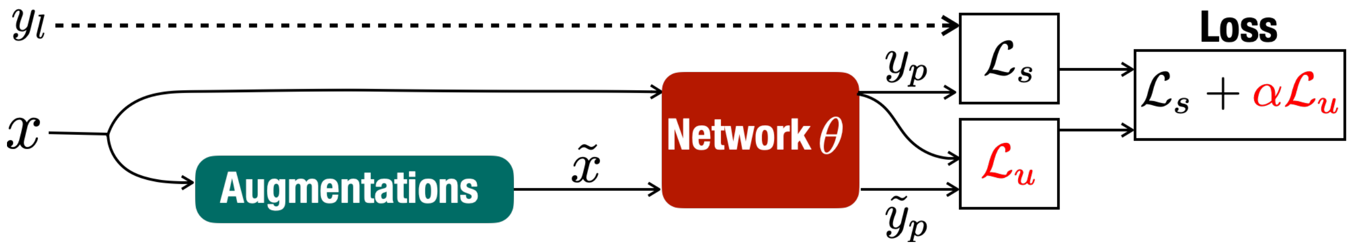

Fig. S2. A simple framework of semi-supervised learning with both supervised ($\mathcal{L}_s$) and unsupervised ($\mathcal{L}_u$) loss functions. The unsupervised loss $\mathcal{L}_u$ is typically used to impose some consistency constraint on the training process.
